# Oral lesions in patients with primary Sjögren’s syndrome. A case-control cross-sectional study

**DOI:** 10.4317/medoral.23254

**Published:** 2019-12-24

**Authors:** Julia Serrano, Rosa María López-Pintor, Mónica Fernández-Castro, Lucía Ramírez, Mariano Sanz, Elisabeth Casañas, Jesús Alberto García, Sheila Recuero, Cristina Bohorquez, Gonzalo Hernández

**Affiliations:** 1 pHD student. Department of Dental Clinical Specialties, School of Dentistry, Complutense University, Madrid, Spain; 2 Department of Dental Clinical Specialties, School of Dentistry, Complutense University, Madrid, Spain; 3 Rheumatology Service, Hospital Puerta de Hierro, Madrid, Spain; 4 Rheumatology Service, Hospital de la Princesa, Madrid, Spain; 5 Rheumatology Service, Fundación Jiménez Díaz, Madrid, Spain; 6 Rheumatology Service, Hospital Principie de Asturias, Alcalá de Henares, Madrid

## Abstract

**Background:**

To evaluate the presence of oral lesions in a group of patients with primary Sjögren’s syndrome (pSS) and compare these results with a matched control group (CG).

**Material and Methods:**

An observational cross-sectional study was conducted. 61 pSS patients (60 women, 1 man, mean age 57.64±13.52) diagnosed according to the American European Criteria (2002), and 122 matched control patients (120 women, 2 men, mean age 60.02±13.13) were included. Demographic and medical data, oral lesions and salivary flow rate were collected.

**Results:**

Compared with the controls, pSS patients were 3.95 more likely to have oral lesions (OR 3.95; 95% CI 2.06-7.58; *p*=0.0001). 57.4% pSS patients presented oral lesions compared to 25.4% in CG. The most common were candidiasis (13.1% vs 2.5%), traumatic lesions (13.1% vs 4.1%), apthae (8.2% vs 0), and fissuration of the tongue (8.2% vs 0.8%). pSS patients with oral lesions had lower salivary flow levels (stimulated and unstimulated), although these differences were not significant. Significant associations were found between the presence of oral lesions and systemic manifestations and history of parotid gland enlargement in pSS patients.

**Conclusions:**

pSS patients suffer more oral lesions than general population and these lesions may aggravate the pSS disease.

** Key words:**Sjögren’s syndrome, oral lesions, oral diseases, oral manifestations, oral disorders.

## Introduction

Sjögren syndrome (SS) is a systemic autoimmune exocrinopathy of unknown aetiology. Different predisposing factors along with several immune-related processes have been described in its pathogenesis, supporting the idea of a multifactorial disease (1). It can occur as primary SS (pSS), when it courses as an isolated disorder or as secondary SS when it appears in association with another systemic autoimmune disease (2). pSS usually emerges in the 4th - 5th decade of life, affecting women more than men, in a proportion 9:1, and its prevalence rates range between 0.1 to 4.8% (1,3,4).

pSS is a chronic inflammatory disease characterized by lymphocytic infiltration of the lacrimal and salivary glands resulting in the primary symptoms of this condition: presence of dry eyes and hyposalivation. Almost half of the patients develop extraglandular manifestations, such as pulmonary, liver, kidney, vascular system, nervous system, respiratory and/or gastrointestinal tract involvement (4). Different pSS classification systems have been published (5), although the most widely used has been the proposed by the American-European Consensus Group (AECG) in 2002 (6). In 2016, the American College of Rheumatology and the European League against Rheumatism developed new classification criteria replacing the previous ones (7).

Saliva is essential in maintaining oral health. It has important functions, such as lubrication, buffering, antibacterial/antifungal activity, as well as the facilitation of digestion and tooth remineralization. The decrease of saliva increases the risk of tooth decay, oral soreness, taste alterations and halitosis. It may also increase the difficulty in wearing dentures. Painful tongue, fissures on the tongue and mucosal ulcers are also common in pSS patients (2,8,9,10,11). Only two studies (10,11) have reported the prevalence of oral lesions in these patients and previous investigations have connected the use of dental prosthesis with the presence of oral lesions, especially candidiasis (9,10,12) but there are no studies that relate other predisposing factors such as tobacco or alcohol consumption, drug intake, or other systemic diseases such as diabetes or hypertension with the presence of oral lesions in pSS patients.

Therefore, the main objective of the present study was to evaluate the presence of oral lesions in pSS patients and to compare with a matched CG. The second goal was to study the association between oral lesions and different risk factors and pSS variables.

## Material and Methods

- Study design and patient selection

A cross-sectional case-control study was conducted, with an inclusion period of twenty months. The present study is part of a project (EPOX-SSp project) studying the oral health condition of pSS patients in the Community of Madrid (12). All the procedures were carried out according to the Declaration of Helsinki and its following revision, and the study protocol was approved by the Ethical Committee of the University Hospital of La Paz, Madrid (no: HULP PI-1891). This study followed the STROBE guidelines for reporting.

A consecutive sampling of all pSS patients attending different rheumatology services in the region of Madrid between October 2015 and June 2017, were invited to participate. These inclusion criteria included being over 18 years old and having the diagnosis of pSS according to the diagnostic criteria proposed by the AECG (6). Subjects were excluded when the selected patient presented physical or psychological difficulties to attend the School of Dentistry or with a history of systemic autoimmune connective tissue disease (apart from pSS). A rheumatologist initially evaluated the inclusion and exclusion criteria and registered the demographic and pSS characteristics, including the time from diagnosis, serological data (rheumatoid factor, immunoglobulins alteration, antinuclear autoantibodies), and systemic manifestations of pSS (parotid enlargement, musculoskeletal, skin, lung, renal, central nervous system, peripheral nervous system, haematological, gastrointestinal or cardiac involvement). Selected patients were then instructed to contact the Oral Medicine Postgraduate Program at the Faculty of Odontology in the Complutense University of Madrid for an oral examination. The matched CG consisted of consecutive patients with similar age and gender, who attended a primary physician consultation for routine medical check-ups at different Health Centers of the Region of Madrid, but not in relation with any oral pathology. Exclusion criteria for CG were patients in treatment with corticosteroids, antifungal or antibiotic agents, and/or with history of systemic autoimmune connective tissue disease.

- Variables and data sources

At the Faculty of Odontology, all demographic data (age and gender), medical history, drugs (type and number of medicines), alcohol intake, tobacco consumption and dose as well as presence and type of dentures were recorded in both groups. A complete oro-facial examination was carried out by a specialist in oral medicine (JS), focused on detecting parotid gland enlargement and intraoral mucosal or oral lesions. To diagnose oral lesions the WHO guidelines were followed (13) recording the number, type of lesion, location, size, clinical appearance, time of evolution and signs and symptoms.

Collection of stimulated (SWS) and unstimulated saliva (UWS) was performed in pSS patients by one specialist in oral medicine (LR). Appointments were between 8.00 and 10.00 am, and patients were asked not to brush their teeth, eat, drink or smoke for at least 90 minutes prior to the appointment. UWS was always collected first by drooling for 15 minutes. Patients were told to be relaxed, and to keep the mouth slightly open and not to swallow. To collect SWS patients were given a paraffin gum and asked to chew it and continuously spit out the saliva into a plastic container for 10 minutes. The flow rates were recorded as ml/min. Hyposalivation was defined as a flow rate <0.7ml/min for SWS and <0.1ml/min for UWS. Additionally, each patient in the pSS group was asked if they had dry mouth sensation, dysphagia and alteration in taste or pain in the tongue.

- Data Analysis. Sample size

The sample size was calculated considering the data on prevalence of oral lesions (61.2%) in pSS patients reported by Likar-Manookin *et al*., Since this study did not compare OLs with a CG, we used the percentage of OLs of the CG of the study of López-*Pi*ntor *et al*., (14) which compared the presence of OLs in renal transplant patients with a CG (23.4%) using the same methodology. Using an α=0.05 and a statistical power of 95%, 41 subjects were needed in each group.

- Data Analysis. Observational Study

Categorical variables were presented as numbers and percentages while quantitative variables as means ± standard deviation (SD). Kolmogorov-Smirnoff test was applied to establish the goodness of fit to normality for the variables studied. To determine differences of categorical variables between the two groups chi-squared test or Fisher’s exact test were used. Differences of quantitative variables between CG and pSS group were compared by the t-student test. U Mann-Whitney test was used to determine the possible relationship between pSS intragroup quantitative variables and the presence of oral lesions. To identify statistically significant association between oral lesions and pSS binary logistic regression was applied. From this analysis, odds ratio (OR) with 95% Confidence Interval (CI) was presented with the associated *p-value*. Differences were considered significant if *p* was ≤ than 0.05.

## Results

All demographic data from the selected patients are depicted in Table 1. Sixty-seven patients diagnosed with possible pSS were evaluated at the oral medicine clinic in the Complutense University. Among them, 61 patients (60 women and 1 man, mean age 57.64±13.52 years) fulfilled the inclusion criteria proposed by the AECG in 2002. One hundred twenty-two CG patients (120 women and 2 men, 60.02±13.13 years) were included.

Table 2 depicts the characteristics of pSS patients, according with the 2002 AECG. pSS systemic manifestations were also recorded and expressed as number and percentage.

- Oral lesions in pSS and CG

Compared with the control group, pSS patients were 3.95 more likely to have oral lesions (OR 3.95; 95% CI 2.06-7.58; *p*=0.0001). A 57.4% of pSS patients had some type of oral lesion vs. 25.4% of CG patients (*p*=0.0001). The mean number of oral lesions was 0.75±0.79 in the study group and 0.27±0.51 in the CG (*p*=0.0001). Twenty-one different types of lesions were recorded. The most common in both pSS and CG were candidiasis (13.1% vs. 2.5%, *p*=0.007), traumatic lesions (13.1% vs. 4.1% *p*=0.03), apthae (8.2% vs. 0%, *p*=0.004), and grooves or fissuration of the tongue (8.2% vs. 0.8% *p*=0.02%). The most frequent type of oral candidiasis was denture stomatitis (8.2% vs. 1.6%, *p*=0.04) (Table 3).

- Oral lesions and clinical pSS variables 

In pSS group, a significant relationship was found between the presence of systemic manifestations associated to pSS, and the appearance of oral lesions. 69.7% of pSS patients with systemic manifestation of the disease suffered oral lesions (*p*=0.03). Additionally, 75% of pSS patients who had had history of parotid enlargement presented oral lesions (*p*=0.05) (Table 2).

- Oral lesions and salivary flow rates in pSS

Table 4 shows the relationship between the presence of oral lesions and UWS and SWS flow rates and hyposalivation. pSS patients with oral lesions suffered more UWS and SWS hyposalivation and had lower UWS and SWS flow rates compared with those without oral lesions, but no significant associations were found.

**Table 1:**
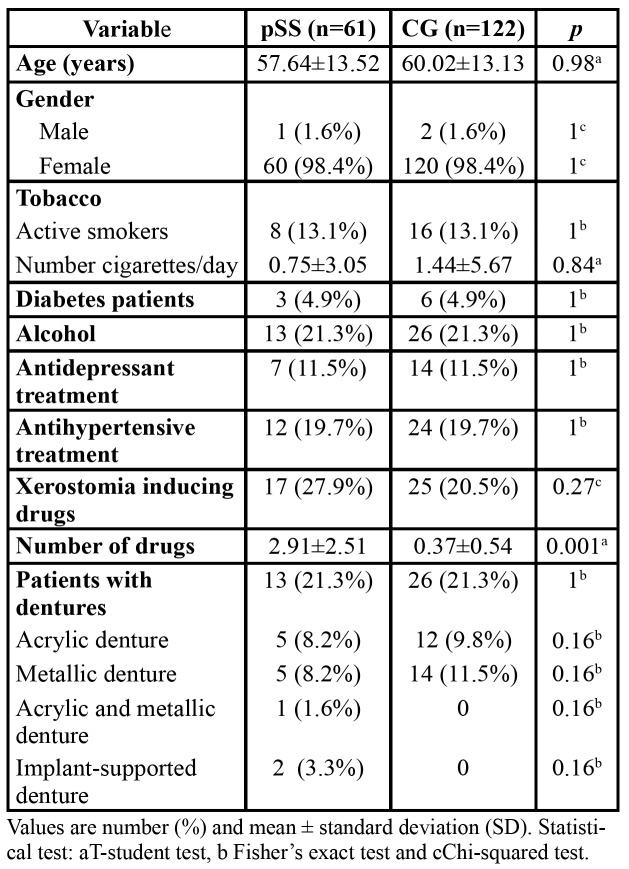
pSS and CG patients demographic data.

**Table 2:**
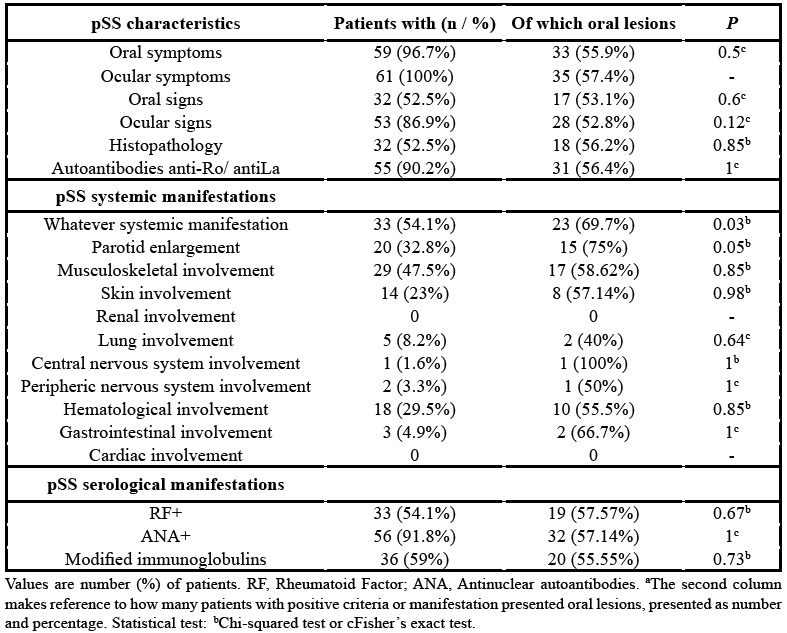
pSS characteristics of the study group (n=61) according with the 2002 AECG and its relationship with oral lesions. pSS systemic and serological manifestations and its relationship with oral lesions.

**Table 3:**
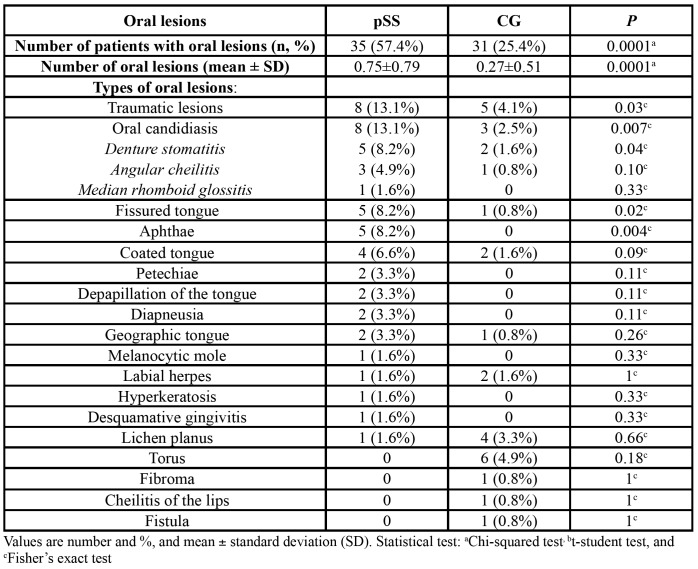
Types and number of oral lesions in pSS and CG.

- Oral lesions as potential risk factors in pSS

No other relationships were observed between the rest of specific pSS variables (including time of diagnosis and onset of oral symptoms) and the presence of oral lesions, neither between other medical or pharmacological features, tobacco, alcohol consumption, or use of prosthesis. All data are collected in Table 4.

**Table 4:**
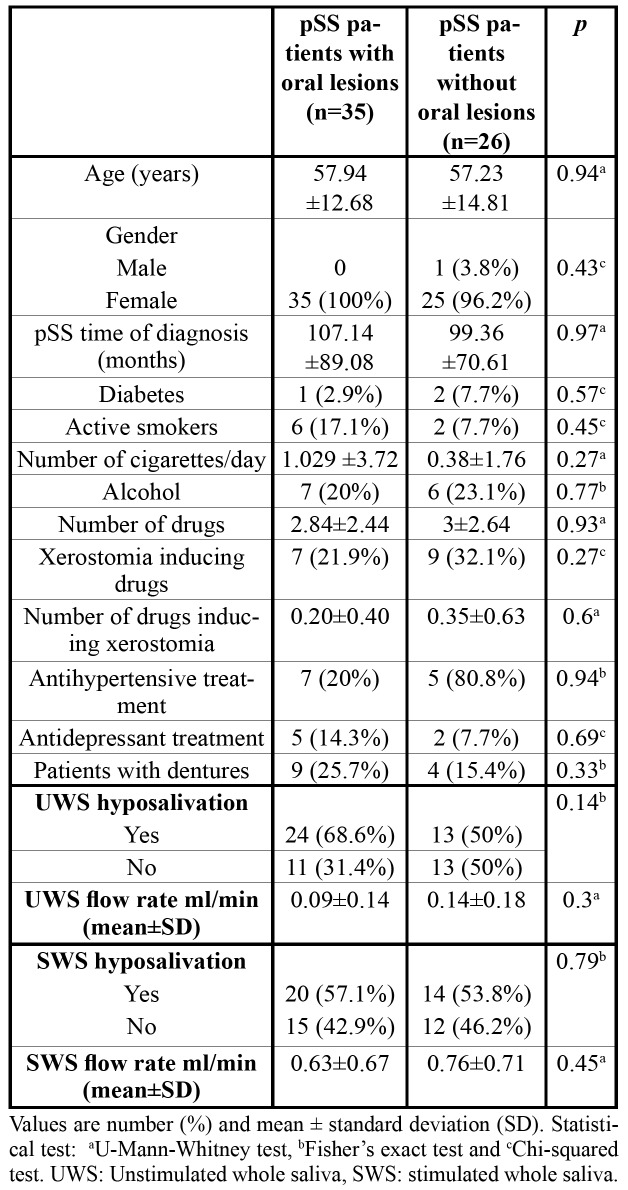
Differences between pSS patients with and without oral lesions. Relationship between hyposalivation, UWS and SWS flow rates and oral lesions.

- Oral lesions and oral symptoms in pSS

The relationship between oral symptoms and the presence of lesions was studied in pSS patients. A 94.3% of pSS patients with oral lesions also suffered dry mouth sensation, 42.3% dysphagia, 37.1% glossodynia and 25.7% alteration in the taste of food. We did not find any statistical significance between having or not oral lesions and symptoms of oral discomfort, although xerostomia (94.3% vs 88.5%, *p*=0.64) and glossodynia (37.1% vs 26.9%, *p*=0.42) was slightly higher in the group with oral lesions.

## Discussion

The present case-control study has shown that pSS patients are more likely to suffer from oral lesions than a matched CG, especially from candidiasis, traumatic lesions, aphthae and fissuration of the tongue. pSS patients with oral lesions had lower salivary flow levels and suffered more systemic manifestations and history of parotid gland enlargement than pSS patients without oral lesions.

SS is known to be one of the most common rheumatic diseases. The lack of saliva could predispose to SS patients to suffer from some types of oral lesions such as coated tongue, atrophic mucosa or grooves or fissuration of the tongue (16). Additionally, people with SS seem to have an increased incidence of fungal infections. The process of *Candida* colonization of oral tissues depends on different factors, including the interaction with salivary proteins. Saliva includes proteins such as histatins (antifungal peptides), thus hyposalivation may not only alter the volume of saliva, but also the oral microflora and increase the risk of opportunistic fungal infections (17,18).

Previous studies have reported the relationship between a reduction of salivary flow rates and **Candida* albicans* counts in saliva (19,20), which sometimes could be associated with clinical signs of candidiasis (11,21,22,23,24). These studies have not established the relationship between salivary flow rates and other oral lesions apart from oral candidiasis. Only Pedersen *et al*., (12) described that oral mucosal changes (and not only candidiasis) occurred more frequently in pSS patients with lower salivary flow rates. To our knowledge, our study is the first to relate UWS and SWS with oral lesions in pSS patients. In our study, UWS and SWS hyposalivation and lower salivary flow rates were more frequently found in pSS patients with oral lesions although there were no statistically significant differences. In this sense, Lynge Pedersen *et al*., found that atrophic tongue and fissured tongue were associated with low UWS rates in a sample of older people in Copenhagen. This finding is in agreement with Bergdhal *et al*., that showed a relationship between the presence of oral lesions in a group of adult women and lower UWS flow rates (25,26).

Until date, only two studies have reported the full prevalence of oral lesions in SS patients. Patinen *et al*., found that 80% of SS patients presented some type of oral lesions compared to 40% of oral lesions in a group of patients with celiac disease. Likar-Manookin *et al*., reported a total percentage of 61.2% of oral lesions in a group of patients with pSS but they did not compare it to a CG (10,11). In our study, 57.4% of pSS patients presented oral lesions vs 25.4% in the CG. In addition, pSS also suffered a higher number of oral lesions compared with CG (0.75 vs 0.27). Our results are more similar to the ones obtained by Likar-Manookin *et al*., than to the results of Patinen *et al*., presumably due to the characteristics of the sample. Furthermore, our findings showed that pSS patients were 3.95 times more likely to suffer oral lesions than the CG. Until date, this is the first study that assessed this association.

A recent systematic review that analyses the presence of oral lesions in SS patients (27) has concluded that the most frequent oral lesions in these patients were angular cheilitis, atrophic glossitis, oral candidiasis, denture stomatitis, traumatic lesions, grooves of the tongue and atrophic mucosa. This is in accordance with what we found in the present study. The most common oral lesions found in pSS were oral candidiasis (13.1%), denture stomatitis (8.2%), traumatic lesions (13.1%), apthae (8.2%), coated tongue (6.6%) and grooves or fissuration of the tongue (8.2%). This systematic review (27), emphasized that the included studies had some limitations, since most of them did not compare with a CG and although some of them collected other potential risk factors (especially smoking habits, use of removable prosthesis and drugs intake) none of them related them to the presence of oral lesions. In this regard, we want to highlight that we took into account every possible local factor that may be related to the onset of any oral lesions of any aetiology, although no significant differences were found. Likar-Manookin *et al*., reported that they did not find any relationship between drugs intake and the presence of autoimmune oral lesions; neither did we in our present study. Some authors suggested that denture wearing should be taken as a predisposing factor for suffering from oral fungal infection (angular cheilitis and denture-induced stomatitis) and oral ulcers in SS patients (24,28), although, other studies (9) did not find significant differences when compared to a CG, as the present study did.

Likar-Manookin *et al*., reported a prevalence of 12% of autoimmune oral lesions in a cohort of pSS patients. They found a prevalence of 7.1% of lichen planus and 3.9% of recurrent aphthous stomatitis. In our study, we have found the same two oral lesions of possible autoimmune aetiology, lichen planus (1.6%) and apthous stomatitis (8.2%) (11). The number of each oral lesion differs between the studies, but as Likar-Mannokin *et al*., suggested we though that pSS and autoimmune oral lesions might have a common aetiology due to a hyperactive autoimmune response. pSS patients with systemic involvement are considered to have a more aggressive type of pSS with a higher risk of morbidity, mortality, cardiovascular and hospitalization risk (29). For this reason, the heterogeneity of SS has been emphasized in the past years, highlighting the importance of the early identification of both glandular and extraglandular manifestations. To date, this is the first study that has evaluated the relationship between oral lesions and different pSS clinical variables and potential predisposing factors such as disease progression or time from diagnosis besides all the possible systemic manifestations. Other authors (14,17,21,23) collected the time from diagnosis and mean duration of the disease, but did not correlate them with the presence of oral lesions. Indeed, we have found that those patients with systemic manifestations and/or previous parotid enlargement had a higher prevalence of oral lesions, which could be related with a higher activity of the disease. In our opinion, patients with pSS systemic involvement should frequently attend to both their medical and dental check-ups, in order to prevent possible oral complications.

Our study has also attempted to find if there was a relationship between oral symptoms and the presence of oral lesions in pSS patients. Almost 95% of pSS patients with oral lesions also suffered from dry mouth sensation, 42.3% dysphagia, 37.1% glossodynia and 25.7% alteration in the taste of food. These results are in accordance with the study of Marton *et al*., who reported xerostomia in 91.8%, glossodynia in 38.7% and dysphagia in 35% of SS patients (9). Although we did not find any statistically significance between having or not oral lesions and oral symptoms of discomfort, xerostomia and glossodynia, the symptoms were slightly higher in the group with oral lesions. Marton *et al*., obtained similar results in their SS patients, 50% of those with candidiasis suffered from glossodyinia as well.

One of the limitations of this study was the number of participants. pSS patients were referred from the different rheumatology services but not all of them attended to the school of dentistry. Another limitation was the study design because our study is a cross-sectional study. It is necessary to carry out future longitudinal studies where patients could be assessed at different times, looking for possible oral lesions, salivary flow rates and oral symptoms, and its possible relationship both with systemic and local factors.

In conclusion, this study shows that pSS patients were more likely to suffer oral lesions than the CG. Oral lesions seem to be more frequent in those pSS patients with systemic manifestations and especially with previous parotid involvement. Due to the heterogeneity and complexity of the disease it is necessary to highlight the importance that pSS patients should be managed by a multidisciplinary team including at least rheumatologists, ophthalmologists and dentists. Dentist should be aware of the risk of suffering oral lesions of these patients, probably related to the lower levels of saliva. Besides, these oral lesions usually do not produce symptoms so frequent check-up appointments are necessary to diagnose them. Future follow-up longitudinal studies with larger number of pSS patients, and where the whole oral lesions are taken into account are necessary to establish the relationship between oral lesions and different features of pSS patients.

## References

[1] Medeiros CCG, Borges LGDA, Cherubini K, Salum FG, Silva RMD, de Figueiredo MAZ (2018). Oral yeast colonization in patients with primary and secondary Sjögren's syndrome. Oral Dis.

[2] Baer AN, Wallit B (2018). Update on Sjögren syndrome and other causes of sicca in older adults. Rheum Dis Clin North Am.

[3] Mavragani CP, Moutsopoulos HM (2010). The geoepidemiology of Sjögren's syndrome. Autoimmun Rev.

[4] Tincani A, Andreoli L, Cavazzana I, Doria A, Favero M, Fenini MG (2013). Novel aspects of Sjögren's syndrome in 2012. BMC Medicine.

[5] Billings M, Amin Hadavand M, Alevizos I (2017). Comparative analysis of the 2016 ACR- EULAR and the 2002 AECG classification criteria for Sjögren's syndrome: Findings from the NIH cohort. Oral Dis.

[6] Vitali C, Bombardieri S, Jonsson R, Moutsopoulos H M, Alexander EL, Carsons SE (2002). European classification criteria for Sjögren's syndrome (SS). Classification criteria for Sjogren's syndrome: A revised version of the European criteria proposed by the American-European Consensus Group. Ann Rheum Dis.

[7] Shiboski CH, Shiboski SC, Seror R, Criswell LA, Labetoulle M, Lietman TM (2016). 2016 American College of Rheumatology/European League Against Rheumatism classification criteria for primary Sjögren's syndrome. A consensus and data-driven methodology involving three international patient cohorts. ARD.

[8] Ship JA, Fox PC, Baum BJ (1991). How much saliva is enough? 'Normal' function defined. JADA.

[9] Márton K, Boros I, Varga G, Zelles T, Fejérdy P, Zeher M (2006). Evaluation of palatal flow rate and oral manifestations in patients with Sjögren's syndrome. Oral Dis.

[10] Patinen P, Aine L, Collin P, Hietanen J, Korpela M, Enckell G (2004). Oral findings in coeliac and Sjögren's syndrome. Oral Dis.

[11] Likar-Manookin K, Stewart C, Al-Hashimi I, Curtis W, Berg K, Cherian K (2013). Prevalence of oral lesions of autoimmune etiology in patients with primary Sjogren's syndrome. Oral Dis.

[12] Pedersen AM, Reibel J, Nordgarden H, Bergem HO, Jensen JL, Nauntofte B (1999). Primary Sjögren's syndrome: salivary gland function and clinical oral findings. Oral Dis.

[13] Fernández Castro M, et al (2019). Evaluación protocolizada odontológica en el paciente con síndrome de Sjögren primario. Proyecto EPOX-SSp: metodología y objetivos. Reumatol Clin.

[14] Kramer IR, Pindborg JJ, Bezroukov V, Infirri JS (1980). World Health Organization. Guide to epidemiology and diagnosis of oral mucosal diseases and conditions. Comm Dent Oral Epidemiol.

[15] López-Pintor RM, Hernández G, de Arriba L, de Andrés A (2010). Comparison of oral lesion prevalence in renal transplant patients under immunosuppressive therapy and healthy controls. Oral Dis.

[16] Blochowiak K, Olewicz-Gawlik A, Polanska A, Nowak-Gabryel M, Kocięcki J, Witmanowski H (2016). Oral mucosal manifestations in primary and secondary Sjögren syndrome and dry mouth syndrome. Adv Dermatol Allergol.

[17] Torres SR, Peixoto CB, Caldas DM, Silva EB, Akiti T, Nucci M (2002). Relationship between salivary flow rates and Candida counts in subjects with xerostomia. Oral Sur Oral Med Oral Pathol Oral Radiol.

[18] Hsu S, Dickinson D (2006). A new approach to Managing Oral Manifestations of Sjögren's Syndrome and Skin Manifestations of Lupus. Biochem Mol Biol.

[19] Tapper-Jones L, Aldred M, Walker DM (1980). Prevalence and intraoral distribution of Candida albicans in Sjögren's syndrome. J Clin Pathol.

[20] Navazesh M (1993). Methods for collecting saliva. Ann N Y Acad Sci.

[21] Koseki M, Maki Y, Matsukubo T, Ohashi Y, Tsubota K (2004). Salivary flow and its relationship to oral signs and symptoms in patients with dry eyes. Oral Dis.

[22] Olate S, Muñoz D, Neumann S, Pozzer L, Cavalieri-Pereira L, de Moraes M (2014). A descriptive study of the oral status in subjects with Sjögren's syndrome. Int J Clin Exp Med.

[23] Rhodus NL, Bloomquist C, Liljemark W, Bereuter J (1997). Prevalence, density, and manifestations of oral Candida albicans in patients with Sjögren's syndrome. J Otolaryngol.

[24] Yan Z, Young LA, Hua H, Xu Y (2011). Multiple Oral Candida Infections in Patients with Sjögren's syndrome- Prevalence and Clinical and Drug Susceptibility Profiles. J Rheum.

[25] Lynge Pedersen AM, Nauntofte B, Smidt D, Torpet LA (2015). Oral mucosal lesions in older people: relation to salivary secretion, systemic diseases and medications. Oral Dis.

[26] Bergdahl M (2000). Salivary flow and oral complaints in adult dental patient. Community Dentistry and Oral Epidemiology.

[27] Serrano J, López-Pintor RM, González-Serrano J, Fernández-Castro M, Casañas E, Hernández G (2018). Oral lesions in Sjögren's syndrome: a systematic review. Med Oral Patol Oral Cir Bucal.

[28] Ergun S, Cekici A, Nursen Topcuoglu N, Migliari DA, Güven Külekçi G, Tanyeri H (2010). Oral status and Candida colonization in patients with Sjögren's Syndrome. Med Oral Patol Oral Cir Bucal.

[29] Ferro F, Vagelli R, Bruni C, Cafaro G, Marcucci E, Bartolini E (2016). One year in review 2016: Sjögren's syndrome. Clin Exp Rheumatol.

